# Meta-Analysis of Differential Connectivity in Gene Co-Expression Networks in Multiple Sclerosis

**DOI:** 10.3390/ijms17060936

**Published:** 2016-06-15

**Authors:** Teresa Maria Creanza, Maria Liguori, Sabino Liuni, Nicoletta Nuzziello, Nicola Ancona

**Affiliations:** 1 Institute of Intelligent Systems for Automation, National Research Council of Italy, 70126 Bari, Italy; ancona@ba.issia.cnr.it; 2 Center for Complex Systems in Molecular Biology and Medicine, University of Turin, 10123 Turin, Italy; 3 Institute of Biomedical Technologies, National Research Council of Italy, 70126 Bari, Italy; maria.liguori@ba.itb.cnr.it (M.L.); sabino.liuni@ba.itb.cnr.it (S.L.); nicoletta.nuzziello@gmail.com (N.N.); 4 Department of Basic Medical Sciences, Neuroscience and Sense Organs, University of Bari, 70126 Bari, Italy

**Keywords:** gene expression, multiple sclerosis, gene networks

## Abstract

Differential gene expression analyses to investigate multiple sclerosis (MS) molecular pathogenesis cannot detect genes harboring genetic and/or epigenetic modifications that change the gene functions without affecting their expression. Differential co-expression network approaches may capture changes in functional interactions resulting from these alterations. We re-analyzed 595 mRNA arrays from publicly available datasets by studying changes in gene co-expression networks in MS and in response to interferon (IFN)-β treatment. Interestingly, MS networks show a reduced connectivity relative to the healthy condition, and the treatment activates the transcription of genes and increases their connectivity in MS patients. Importantly, the analysis of changes in gene connectivity in MS patients provides new evidence of association for genes already implicated in MS by single-nucleotide polymorphism studies and that do not show differential expression. This is the case of amiloride-sensitive cation channel 1 neuronal (*ACCN1*) that shows a reduced number of interacting partners in MS networks, and it is known for its role in synaptic transmission and central nervous system (CNS) development. Furthermore, our study confirms a deregulation of the vitamin D system: among the transcription factors that potentially regulate the deregulated genes, we find *TCF3* and *SP1* that are both involved in vitamin D3-induced p27Kip1 expression. Unveiling differential network properties allows us to gain systems-level insights into disease mechanisms and may suggest putative targets for the treatment.

## 1. Introduction

Multiple sclerosis (MS) is a chronic and progressive autoimmune disease characterized by demyelination and degeneration within the CNS. The MS etiology is still complex and mostly unclear, with genetic and environmental factors possibly influencing the susceptibility and/or the outcomes of the disease. Several variables associated with latitude, e.g., the daily vitamin D intake, have been investigated as possible influencing factors [1]. On the other hand, 15%–20% of the patients reported a family history of the disease, thus confirming the (although modest) heritability of MS [1]. Genes located within the Human Leukocyte Antigen (HLA) region are the most important MS susceptibility loci [2,3], but several international genome-wide association studies (GWAS) studies have led to the identification of other candidates for the disease susceptibility [4,5,6]. However, at present, some other unidentified genes, potential interactions between genetic and environmental factors, as well as the influence of epigenetic factors able to modulate their expression still leave unsolved questions of the pathogenic issue of MS. Particular attention has been recently devoted to the study of gene expression by using microarray technology (and more recently, RNA-sequencing), thus supplying useful information on the different MS courses and phenotypes [7,8]. In detail, these studies analyzed those genes that exhibited a significant change of their expression levels between two different phenotypic conditions and highlighted putative signals associated with the MS onset and progression. Although differential gene expression studies have been widely and successfully applied, they present severe drawbacks. It is well known that genetic mutations can occur in both regulatory or coding regions of genes. Moreover, mutations in coding regions and post-translational modifications (e.g., phosphorylation, methylation, acylation, *etc*.) can influence the regulatory activity of a gene without affecting its expression level, but altering the expression profiles of its interacting genes [9]. Therefore, an analysis based uniquely on differential expression approaches that considers genes individually could be ineffective for the purpose of highlighting key genetic drivers in complex diseases. On the other hand, networks based on gene expression pairwise correlations can represent direct gene regulations and also include genes that are indirectly connected through regulatory pathways [10] in a condition-specific manner. For instance, in relation to single nucleotide polymorphisms (SNPs) associated with a given disease, the work of Fairfax *et al.* suggests that certain risk alleles can alter the correlated gene expression profiles of specific genes in a context-specific manner, possibly removing such genes from their normal regulatory influences [11]. In this context, a new trend is emerging in transcriptomic studies in which statistically significant changes in correlations between gene pairs are detected in order to reveal changes in functional interactions and dependencies or coordinated activities of genes in response to specific conditions and perturbations [12]. Differential mapping approaches have been applied to the study of complex diseases with the aim to identify changes in gene interaction patterns, since they can be linked directly to differences in molecular mechanisms underlying the disease pathogenesis. These network approaches, including differential co-expression analysis (DCA), have shown success in the investigation of cancer gene networks [12,13], as well as in the study of the response of human cells to different stresses, such as endoplasmic reticulum stress and exposure to ionizing radiation [14]. Moreover, Rhinn *et al.* [15] analyzed the influence of the presence of the apolipoprotein E *ϵ*4 allele (APOE4) on the late-onset Alzheimer’s disease (LOAD) risk by using DCA approaches with the aim to identify a set of candidate core regulatory mediators. The authors clearly remark that DCA is based on the notion that transcripts encoding causal nodes or master regulators of a pathological process, whose activities are critically altered in a pathological state, such as LOAD, can be identified by their co-expression network properties, but not by simple differential gene expression analysis. Their work suggests as potential causal nodes those transcripts having altered co-expression correlations with the greatest number of differentially-expressed transcripts.

**Figure 1:**
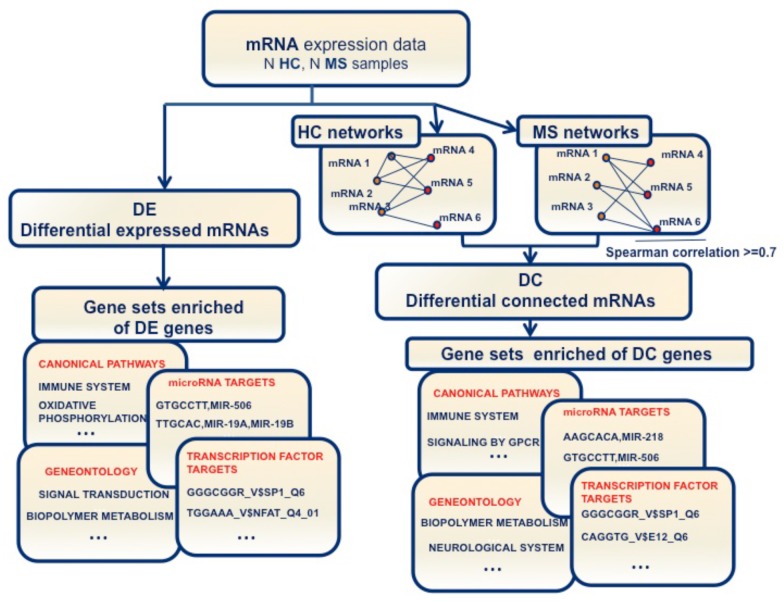
Bioinformatics workflow of the study. The picture refers to the MS *vs.* HC comparison. The same workflow was adopted to analyze datasets related to interferon (IFN)-β-treated and untreated MS patients.

All of these considerations motivated the study of both the rewiring of gene co-expression networks in MS with respect to the healthy condition and of networks inferred in untreated patients *vs.* those related to patients treated with some of the most commonly-used drugs in MS. Encouraged by the success of differential connectivity approaches in the studies of cancer in identifying putative driver genes [12], we applied these methods to analyze the changes in gene co-expression networks occurring in MS. Figure 1 depicts the workflow adopted in the study. In detail, by comparing gene co-expression networks related to blood samples of MS patients *vs.* healthy controls (HCs), we searched for genes exhibiting the greatest changes in their co-expression patterns in MS patients. Moreover, datasets related to interferon (IFN)-β-treated and untreated MS patients were analyzed in order to unveil how this commonly-used immunomodulatory treatment affects the gene interaction patterns. This paper aims to complement and integrate previous studies that analyze gene expression data in whole blood from MS patients with the relapsing-remitting (RR) form of the disease and HCs by applying, besides the standard differential expression (DE) tests, methods based on differential gene connectivity (DC). Moreover, enrichment analyses of the Gene Ontology terms and the canonical pathways performed on the DE and DC gene lists help to understand which are the deregulated mechanisms underlying the disease onset and progression. Finally, enrichment analyses of transcription factor and microRNA targets applied on the lists of the deregulated genes suggest potential upstream regulators that are responsible of their changes in expression and connectivity.

## 2. Results

### 2.1. Changes of Connectivity in MS Networks

We studied the gene interaction changes that emerged in MS patients relative to HCs by comparing the specific inferred co-expression networks. To this end, we analyzed three independent datasets (see Table 1) retrieved from the Gene Expression Omnibus (GEO) database [16].

**Figure 2:**
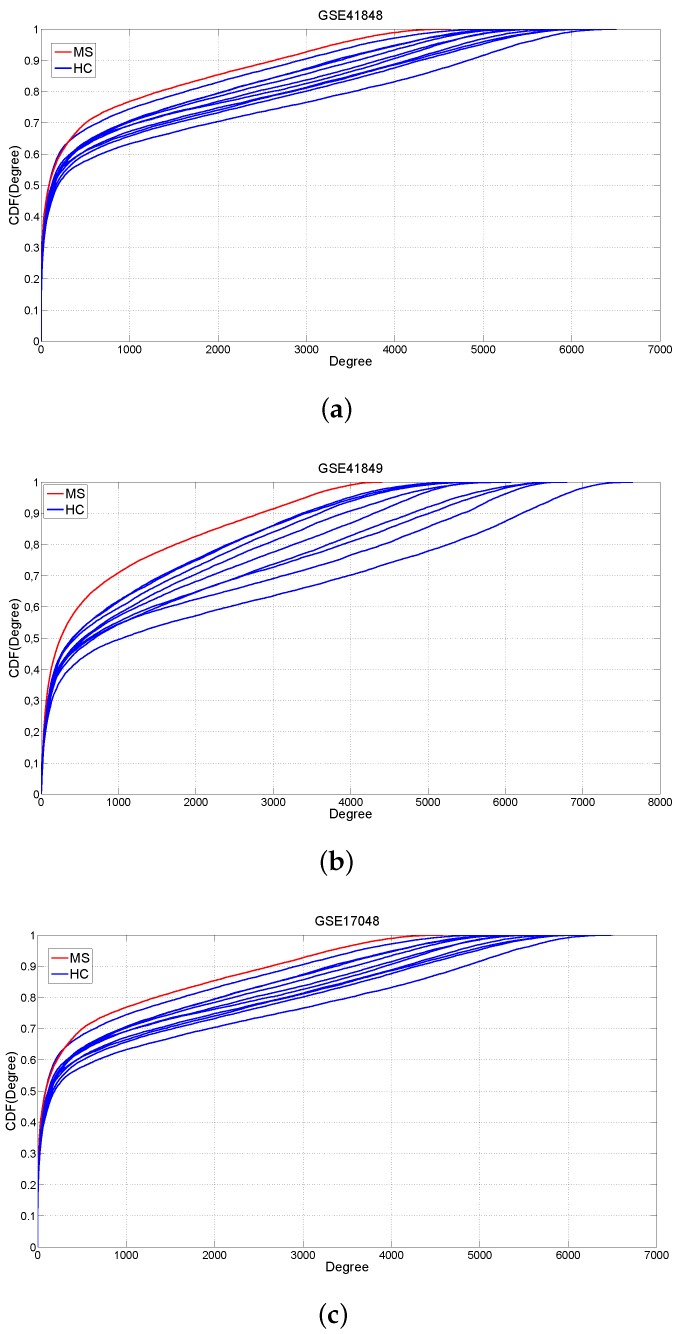
Cumulative distribution functions of the gene degree. Comparison between HC (blue color) and MS (red color) specimens of whole-blood by using the GSE41848, GSE41849 and GSE17048 GEO datasets. (**a**) GSE41848; (**b**) GSE41849; (**c**) GSE17048.

Once inferred the networks in the healthy and diseased states, we investigated their topological properties in terms of gene connectivity. Next, we applied approaches of differential networking by searching for differentially-connected (DC) genes as nodes characterized by a significant degree difference between the two phenotypes (see Materials and Methods). MS and normal networks were characterized by similar topological behaviors (Figure 2): both of them were composed of nodes with highly variable degrees, from genes with a few connections to “hubs” with thousands of links. Importantly, we found a loss of connectivity in MS networks with respect to HCs in all datasets (Figure 2). Indeed, a Kolmogorov–Smirnov test showed that all MS networks were characterized by a gene degree that stochastically decreased with respect to the corresponding HC graphs: $p=10^{-6}$ GSE41848, $p=10^{-64}$ GSE41849 and $p<10^{-100}$ GSE17048 are the maximum *p*-values among the 10 experiments for each GEO study [16].

**Figure 3:**
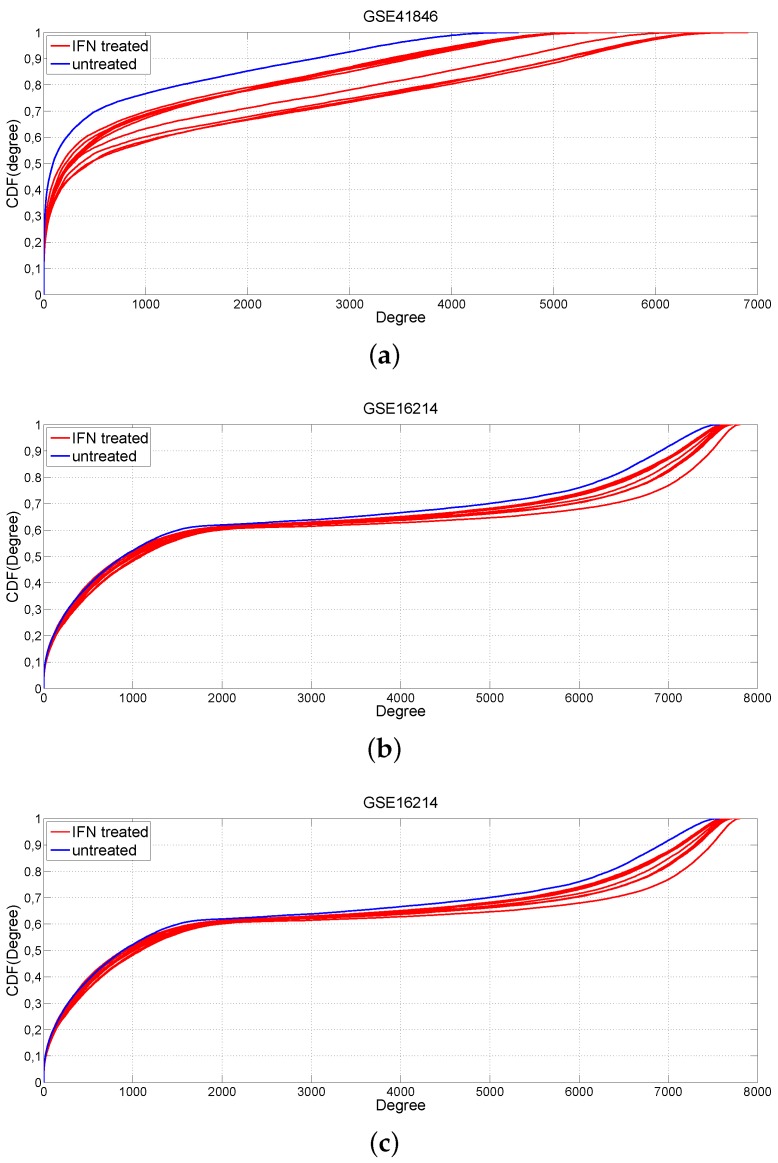
Cumulative distribution functions of the gene degree. Comparison between IFN-treated (blue color) and untreated (red color) MS patients by using the GSE41846, GSE41847 and GSE16214 GEO datasets. (**a**) GSE41848; (**b**) GSE41847; (**c**) GSE16214.

The same analysis was performed for comparing IFN-β-treated with untreated MS patients. MS patients presented an increased connectivity when treated with IFN-β (maximum Kolmogorov–Smirnov test *p*-values $p=10^{-48}$ GSE41846, $p=10^{-15}$ GSE16214; Figure 3a,c) in the two more numerous datasets. For the dataset GSE41847, this result is not confirmed in all comparisons corresponding to the different random sub-samplings (Figure 3b) due to the limited sample size. However, the three studies show that IFN-β treatment restores the overall gene connectivity of MS co-expression networks as confirmed by the Fisher’s *p*-value $p<10^{-100}$ obtained combining the Kolmogorov–Smirnov tests on the three independent studies. The gain in connectivity in response to the interferon-β treatment provided encouraging evidence for the effects of this treatment on MS transcriptomes.

### 2.2. Differential Gene Expression and Connectivity

This preliminary evidence of changes in network connectivity motivated a detailed analysis for each gene, not only in terms of differential expression, but also in terms of modifications of connectivity with its network neighbors. In detail, non-parametric tests were adopted in order to highlight genes with degree variations associated with the pathology and/or treatment and not due to chance (see the Materials and Methods). Consequently, for each gene, we evaluated the DC and DE scores with their *p*-values. Our results are in accordance with previous studies that showed that the number of genes with altered interacting partners was less than the number of DE genes [12,14] (except for the GSE17048). As a consequence, in order to get comparable numbers of DC and DE genes, we decided to adopt a more stringent criterion for the significance level of DE *p*-values: in particular, a threshold of 0.01 for DE *p*-values and 0.05 for DC analyses (Table 2).

The Supplementary Tables S1–S4 report the lists of genes that result in DE or DC in at least two of the three analyzed datasets for the two comparisons MS *vs.* HC and IFN-treated *vs.* untreated MS patients. The tables report the *p*-values and the statistics of differential expression and differential connectivity in each study, as well as a combined *p*-value that is the maximum among the two smallest *p*-values over the *p*-values from the three studies. The tables collect the genes that are DC or DE at the level of 0.05 and of 0.01, respectively, in at least two studies, *i.e.*, genes that have a combined *p*-value that is less than or equal to the fixed significance threshold. Note that these tables are reporting only the genes that are annotated on the different platforms. Of interest, in the case of the MS *vs.* HC comparison, none of the genes DC in at least two experiments results in DE in at least two experiments. This suggests that the analysis of gene expression data focusing on changes in connectivity may reveal genes implicated in MS not highlighted by differential expression analyses on the same data.

### 2.3. DE and DC Pathway Analysis

Encouraged by the the evidence of complementarity between differential expression and connectivity on single genes, we considered that our approach of differential networking might be innovative also in the evaluation of putative pathways involved in MS pathogenesis. To this aim, we explored the biological significance of genes resulting in DC or DE between the different MS conditions by performing an enrichment pathway analysis. In particular, we searched for overrepresented biological functions, molecular mechanisms or pathways that the DE and DC genes are involved in, utilizing the gene sets of the canonical pathway collection (C2CP) and of the Gene Ontology collection from MsigDB (Molecular Signature Database) [21]. In Tables S5–S8 are reported the pathways enriched with DC or DE genes in at least one study with a score that is computed as the sum of the $-log_{10}$(*p*-value) across the different studies.

### 2.4. Analysis of Enriched Transcription Factor and MicroRNA Targets

Among the genetic regulators, transcription factors (TFs) and microRNAs (miRNAs) are the essential key players for regulating gene expression. Some evidence suggests transcription factors and microRNAs as transcriptional regulators of genes involved in MS onset and in cellular reactions to IFN-β stimulation [8,22,23]. A genome-wide analysis of the potential upstream regulators that are responsible for changes in gene expression and connectivity could be useful to shed light on key driver genes and miRNAs in human diseases and, in turn, to suggest novel strategies in the disease treatment. To address this issue, we performed an enrichment pathway analysis to discover upstream regulators of the DE and the DC genes. In detail, we searched for the transcription factor binding sites (TFBSs) in the promoter regions and microRNA binding motifs in the 3’-UTR overrepresented in the DE and DC gene lists. The resulting lists of the TFBSs and microRNA binding motifs enriched with DE genes are reported in the Tables S5 and S7 for the comparisons of MS *vs.* HC and untreated *vs.* IFN-β treated, respectively, in at least one study. Tables S6 and S8 collect the TFBS and the miRNA motifs enriched with DC genes in the comparisons of MS *vs.* HC and untreated *vs.* IFN-β treated, respectively, in at least one study. For each element, a score is shown that is computed as the sum of the $-log_{10}$(*p*-value) across the different studies.

## 3. Discussion

A number of differential gene expression studies at both the individual gene and biological pathway level have been performed to investigate MS molecular pathogenesis and the molecular effects of IFN-β treatment. In this framework, genetic and epigenetic modifications associated with MS can modify the functions of a given gene without modifying its expression, but eliciting variations to expression levels of the regulated genes. For example, a co-signaling molecule in a T-cell can interact with more than one counter-receptor or ligand and, consequently, may either activate or inhibit immunological functions according to a specific molecular state [24]. Consequently, the identification of disease-associated genes requires studying, besides DE genes, genes involved in changes of the functional interactions resulting from the aforementioned modifications. To address this issue, we investigated the rewiring of MS gene interaction networks in terms of differential connectivity in co-expression networks. Our study aims to complement and integrate current studies that analyze gene expression data in whole blood from MS with a specific clinical form (relapsing-remitting (RR)) patients and healthy controls and in response to IFN-β treatment in MS by using differential gene connectivity methods. As a general gene trait, a loss of connectivity results in MS patients when compared to HCs. This evidence suggests that proper physiological functions depend on coordinated gene transcriptions that are impaired in pathological conditions. Given that alterations in functional interactions as a consequence of variants implicated in disease may be investigated also upon triggering of immune responses [11], we analyzed the changes in co-expression networks also in response to IFN-β treatment. It results that this immunomodulatory drug stimulates the transcription of genes and increases their gene functional connectivity in MS patients; in other words, they acquire new interacting partners to engage in IFN-β-specific responses.

### 3.1. Comparison of MS vs. HCs

Out of 1520 DE genes in the GSE41848 dataset, 540 resulted in being confirmed as DE in the validation set GSE41849 (significant overlap according to a one-side Fisher’s exact test $p<10^{-100}$). Only 12 genes were confirmed as DE also in the GSE17048 study (Table S1): of interest, among them, we found three genes upregulated coding for mitochondrial ribosomal proteins (*MRPS24*, *MRPL21*, *MRPL51*) and genes involved in respiratory electron transport, ATP synthesis by chemiosmotic coupling and heat production by uncoupling proteins (*NDUFB7*, *NDUFA13*, *ATP5I*). The two subunits of mitochondrial complex I *NDUFB7* and *NDUFA13* are associated with Alzheimer’s, Parkinson’s and Huntington’s diseases [25].

Concerning the connectivity analyses, 29 genes resulted in DC for at least two of the analyzed datasets. Notably, all of the DC genes lost connectivity in MS with respect to the HC networks (Table 3 and Table 4, Table S2).

This enhances the evidence reported in the previous section of the overall loss of connectivity in MS samples. In particular, we observe a general trait of all genes that present a significant change in their connectivity: all of them exhibit a decreasing degree in MS networks.

Several genes that resulted in DC between MS and HC subjects have been found to be implicated in the pathogenesis of CNS diseases (see Table 3 and Table 4). Some of them have been already reported as connected with MS by studies of SNPs and are genes that do not show a differential expression in MS. In particular, we compared the lists of DE and DC genes with the genes resulting in being associated with MS by the GWAS study in [41]. Gene *p*-values were computed by analyzing the SNP *p*-values of the GWAS study in [41] (see the Material and Methods section). Interestingly, eight out of the 29 DC genes have a *p*-value ≤0.006, and a Fisher’s exact test (*p*-value = 0.045) shows that the list of DC genes is significantly enriched with genes implicated in MS by this GWAS study (see Table S2). Moreover, 23 out of the 227 DE genes are associated with MS by the GWAS study, but this overlap is not significant (Fisher’s exact test *p*-value = 0.99). To demonstrate that the result of the significant overlapping between the list of the DC genes and the list of the genes resulting in being associated with MS by the GWAS analysis is general, we added a statistical analysis that does not require fixing an arbitrary threshold. In detail, a *t*-test shows that the mean of the GWAS *p*-values of the DC genes is significantly smaller than the GWAS *p*-values of the entire list of genes on the chip (one-tail *t*-test *p*-value = 0.05). Moreover, the DE genes have no GWAS *p*-values significantly smaller than the GWAS *p*-values of the entire list of genes (one-tail *t*-test *p*-value = 0.99).

Among the most significantly differentially-connected genes confirmed in all three studies, we found poly(A)-binding protein-interacting protein 1 (*PAIP1*) that stimulates translational initiation by inducing the circularization of mRNA [42]. Deregulation at this step of the translation process leads to altered gene expression, which in turn modifies cell growth. Of interest, the Paip1-eIF3 interaction is impaired by the mammalian target of rapamycin 1 (mTORC1) inhibitors, and activation of mTOR is essential for oligodendrocyte differentiation [43]. Moreover, *PAIP1* was reported to be highly expressed in microglial cells of familial amyotrophic lateral sclerosis [29]. Genes DC in at least two studies include many genes previously-associated with other neurodegenerative disorders, such as the genes *FH*, *UBE2H* and *DLG1* that were found involved in Parkinson’s disease [28,36,44], *KHDRBS2* recently implicated in Alzheimer’s disease [39], the gene *PRKAR2A* in Lewis body disease [37] and the *TCP1* gene in Huntington’s disease [45].

Several DC genes are related to chromatin structural and epigenetic regulation; among them, the histone-lysine *N*-methyltransferase *MLL4*, the *HIST2H2AB* gene encoding a member of the histone H2A family and the gene histone deacetylase 2 (*HDAC2*). Note that almost all of these DC genes did not result in DE in the analyzed datasets, except for the *HIST2H2AB* gene, recently emerging as most likely regulated during the processes of memory consolidation [46] (see Table S2).

### 3.2. Comparison of IFN-β-Treated vs. Untreated MS Patients

Out of 1471 DE transcripts in the GSE41846 dataset, 389 were confirmed in the GSE41847 dataset ($p<10^{-100}$ one-side Fisher’s exact test) (Table S3). The genes that result in DE from the analysis of the GSE41846, GSE41847 and GSE16214 datasets are 187; among them, only 22 (12%) are downregulated. Note that the difference in gene expression for the DE genes is significantly greater than zero for each dataset (the maximum *t*-test *p*-value $p=10^{-23}$). Concerning the network analysis, only 15 genes were DC for all datasets (Table 5, Table S4).

Although this intersection was not statistically significant, it is worth pointing out that 14 out of 15 DC genes exhibited a concordant behavior: they show a higher degree in IFN-β-treated networks. Both the DC and the DE measure showed an overall tendency for all of the deregulated genes, thus suggesting that the administered immunomodulatory drug activates the transcription of genes and increases their gene functional connectivity in MS patients; in other words, they acquire new interacting partners to engage in IFN-β-specific responses. Among the most significantly DC genes, it is worthy mentioning the *XIAP*-associated factor 1 (*XAF1*) gene, which has been already associated with the response to IFN-β treatment in MS patients [58], also leading to the publication of a patent that stated its validity as a marker of IFN-β responsiveness [63]. In the same direction, another DC gene was myxovirus (influenza virus) resistance 1 (*MX1*) (p=0.013 in GSE41846, p=0.029 in GSE41847), one of the first investigated genes also frequently used in clinical practice as a marker of IFN-β responsiveness [64].

Overall, 29% (183) of the genes DE in at least two studies are known IFN-β-responsive genes, as recorded in the Interferome database (last accessed October 2015), and 57% (37) of the genes with a significant change of connectivity in at least two studies are also reported as IFN-β-responsive genes, like *IFI27*, *MX1*, *IFI44L*, *IFIT2*, *XAF1*, *ISG15*, *SAMD9L*, *LGAL9*, and many others [65]. This evidence indicates that a more robust signature of IFN-β-responsiveness can be reliably detected from whole-blood total RNA by our differential network approach with respect to the application of differential expression methods.

In the same direction, the recovered connectivity significantly pointed out known genes involved in the MS inflammatory cascade and the overall network of autoimmunity (*AIM2*, *CXCL10*, *HERC5*, *IFI44L*, *ISG15*, *OASL*, *SUGT1*, *TRIM5*); among them, the *AIM2* gene, encoding an important inflammasome component that senses potentially dangerous cytoplasmic DNA, leading to the activation of the *ASC* pyroptosome and caspase-1 and to pyroptotic neuronal cell death [66]. According to our findings, recent studies suggested a potential modulation of *AIM2* inflammasome activity mediated by IFN-β in MS patients [67].

### 3.3. Enrichment Pathway Analysis

Furthermore, our DC pathway analysis confirms and extends the informative content provided by differential expression: besides several known deregulated pathways, we observed differential connectivity for candidate MS-related biosystems not yet fully investigated. Specifically, our analysis on the lists of DC genes confirms the results provided by DE analyses in [17] by hinting to a role for immune processes in MS and as response to IFN-β treatment (Reactome Immune System $p=10^{-27}$ in GSE41846, $p=10^{-9}$ in GSE41847, $p=10^{-15}$ in GSE16214, $p=10^{-6}$ in GSE41848, $p=10^{-5}$ in GSE41849). Moreover, both DC and DE analyses in the MS *vs.* HC comparison point to genes involved in other neurodegenerative diseases, as well as in oxidative phosphorylation, the TCA cycle and respiratory electron transport. The analysis of the GSE17048 dataset suggests a loss of gene connectivity in MS of genes involved in the functioning of the neuronal systems and, in particular, of neuroreceptors that transmit the signals from the post-synaptic membrane to the cell body. Moreover, this study suggests a loss of gene connectivity also for genes encoding for extracellular matrix proteins (matrisome) [68], known regulators of cell proliferation, survival, differentiation and migration. The DC pathway analysis between MS patients and HCs complements the differential expression analysis, suggesting a role for the G-protein-coupled receptor (GPCR) signaling and the process of megakaryocyte development and platelet production. The comparison between the connectivity of IFN-β-treated *vs.* untreated networks confirms a deregulation of pathways representing the interferon signaling, such as Reactome Interferon signaling and Reactome Interferon α/β signaling (maximum *p*-value $p=10^{-10}$ across the three datasets). Moreover, we performed a genome-wide analysis of the potential upstream regulators that are responsible for changes in gene expression and connectivity in order to reveal key transcription factors and miRNAs in MS.

### 3.4. Overrepresented 3’-UTR MicroRNA Binding Motifs

The lists of the DC and DE genes in both comparisons (MS *vs.* HC and IFN-β treated *vs.* untreated) result in being enriched in microRNA targets of miR-506, miR-17-5P, miR-20a, miR-106a, miR-106b, miR-20b, miR-519d, miR-218, miR-124a, miR-9, miR-145, miR-142-5p, miR-29, miR-27, miR-23, miR-30, miR-524 and miR-98. Of interest, many of these miRNAs (miR-506, miR-524, miR-29A, miR-29B, miR-29C and miR-124a) result also by the analysis of the pathways overrepresented among the 63 non-MHC genes associated by GWAS with MS [69] (see Tables S5–S8). In addition, an enrichment pathway analysis on the entire set of targets of these miRNAs indicates the axon guidance pathway as one of the most frequently-targeted pathways (by the mSigDBenrichment tool). This confirms the involvement of axon guidance proteins in MS and suggests that miRNA-based strategies might be used to address disease-associated changes in neuronal connectivity caused by the altered expression of these proteins. In particular, the lists of DC and DE genes are enriched in targets of miR-506, which is located in Xq27.3; recently, Arora *et al.* [70] have shown that *NF-kB* binds to the upstream promoter region of this miRNA to suppress its transcription. This link seems interesting since *NF-kB* is essential for the peripheral immune cell activation and the induction of autoimmune diseases, like MS [71]. Above all, our findings are consistent with those reported by studies of miRNA differentially expression in whole blood samples from MS patients with respect to HCs. By analyzing miRNA expression profiles (confirmed by RT-PCR), Cox *et al*. have found that miR-17 and miR-20a are significantly under-expressed in MS and have demonstrated that these miRNAs modulate T cell activation genes in a knock-in and knock-down T cell model [72]. Likewise, Keller *et al.* [73] have identified in the blood of MS patients a set of micro-RNAs, including miR-145, miR-20b, miR-142-3p, miR-30a and miR30e, which accurately differentiate patients with RRMS from HCs. In addition, Noorbakhsh *et al.* include miR-142-5p, miR-98, miR-218 and miR-9 in the list of miRNAs whose expression is altered (at least 1.5-fold) in MS brains [74]. Many of the reported overrepresented miRNA targets are known for their functional implications in CNS development and/or associated with MS. Among them, miR-218, enriched with targets in DC and DE MS-associated genes in all three studies, plays a crucial role in motor neuron differentiation [75]. In addition, a pathogenic role of miR-124 is supported by the evidence that this miRNA promotes microglia quiescence; data have shown that peripheral administration of miR-124 in EAE (Experimental autoimmune encephalomyelitis) causes systemic deactivation of macrophages, reduced activation of myelin-specific T cells and marked suppression of the disease [76]. Furthermore, hippocampal demyelination and memory dysfunction have been associated with increased levels of miR-124 and reduced AMPA receptors in postmortem MS brains [77]. Our analysis enlightens several other miRNAs that previous studies have reported to be associated with MS: miR-23B, involved in MS and other autoimmune diseases by suppressing IL-17-associated inflammation that targets the proteins TAB2, TAB3 and IKK-*α* [78]; miR-29a/b, the deficiency of which identifies a negative feedback loop controlling Th1 bias that is dysregulated in MS [79]; and miR-27B, which is up-regulated in memory CD4(+) T cells from patients with MS, inhibiting Th2 cell development and favoring pro-inflammatory Th1 responses [80]. Crosstalk between members of the miR-30-5p family and the canonical Wnt/β-catenin pathway that is involved in MS remyelination is also documented [81,82].

### 3.5. Overrepresented Transcription Factors Binding Sites

Many of the transcription factor binding sites (TFBSs) enriched in both the DC and the DE lists confirm previous research (see Tables S5–S8). Among them, the most significant transcription factors potentially dysregulated in MS are *YY1* (yin and yang 1), *E2F-1/DP-1* and *E2F-4/DP-2* heterodimers, *EGR* family members and *CREB* and *ATF* families. In particular, the *YY1* transcription factor seems to play a key role in the pathogenesis of MS or its subtypes, given its involvement in processes affecting myelin protein generation, viral replication and immune response. In the study of Riveros *et al.* [83], dysregulation by *CREB*/*ATF*, *E2F* families, *ELK-1* and, principally, by *MYB* and *YY1* is associated with MS overall, while *E2F-1*/*DP-1* and *E2F-4*/*DP-2* heterodimers, *NF-kappaB* (p65) and *TTF-1* are specific for RRMS. TFBSs overrepresented in the regulatory regions of the DC and DE genes in the comparison of IFN-β-treated *vs.* untreated patients include IFN regulatory factors, interferon-stimulated response elements, *AP4* and *NF-kB*. Interferon regulatory factors (IRFs) are known transcription factors induced by type I interferons (IFN-α/β). Some studies suggest a cooperative interaction of IRFs with *AP-1* and *NF-kB* at the IFN-β enhancer, in order to contribute to IFN-β transcriptional activation [84]. In particular, IRF7 signaling is critical for the regulation of inflammatory responses in the CNS [85]. TFBSs overrepresented in the regulatory regions of DC and DE genes in both comparisons MS *vs.* HC and IFN-β-treated *vs.* untreated patients share at the top-ranked position the motif GGGCGGR, which matches the annotation for *SP1*. This finding is of particular interest, as Menon *et al.* have reported that specific targeting of *SP1*-dependent gene transcription in peripheral blood mononuclear cells by the inhibitor WP631 impaired T cell responses *in vitro* and *in vivo* and reduced the incidence and severity of experimental autoimmune encephalomyelitis [86]. In addition, the study of Kristjansdottir *et al.* has shown that an increased amount of transcription factor *SP1* binds to the risk allele (the 4×CGGGGallele) of the CGGGG indel polymorphism, which showed evidence of association with MS [87]. From a functional point of view, it is known that the Sp1 family of transcription factors is implicated in the p27Kip1-mediated activation of myelin basic protein gene expression [88]. *SP1* is also known to modulate vitamin D receptor (*VDR*) binding, and there is some evidence that interactions between *SP1* and *VDR* may enable the modulation of genes that lack a classical *VDR* recognition motif [89]. The list of the transcription factor binding sites overrepresented in DC and DE genes includes also the motif CAGGTG that matches with the *VDR*-interacting repressor (*VDIR*) gene. It is interesting to note that also *VDIR* and *VDR*, as *SP1*, have a role in the transcriptional regulation of the *CYP27B1* gene (that encodes for p27Kip1), as ligand-induced association between *VDR* and *VDIR* results in ligand-induced repression (transrepression) of the *CYP27B1* gene expression. This transrepression is associated with *VDIR* switching from a co-activator complex containing histone acetyltransferase (HAT) to a co-repressor complex containing histone deacetylase (HDAC) [90]. Note that many DC genes are epigenetic regulators, and among them, we find *HDAC2*, which is known to physically interact with *VDIR*; this binding is a critical step for chromatin structure remodeling in suppression of the *CYP27B1* gene. Considering that this gene has been deeply investigated as most likely involved in the MS risk, although with conflicting results [91], we believe that the reported data about its regulation may add further insights into the evaluation of vitamin-D-related issues.

Concluding, we noted a convergence of our findings in terms of DC genes and transcription factors regulating them, as *SP1* and *VDIR*, that indicates that in MS, the epigenetic regulatory system is disturbed and that epigenetic modifications affect the regulation of the vitamin D system. In detail, many DC genes are epigenetic regulators and, among them, we find *HDAC2*, which is known to physically interact with *VDIR*, and this binding is a critical step for chromatin structure remodeling in suppression of the *CYP27B1* gene. Our reported evidence that the *HDAC2* gene functions resulted in being impaired in MS is very interesting, since the gene is known to negatively regulate memory formation and synaptic plasticity [92], as well as promoting cellular differentiation and myelination in the majority of oligodendrocytes [40].

Of interest, we found that the list of DC genes is significantly enriched with genes implicated in MS by the GWAS study in [41], but there is not a significant overlap between the DE genes and the GWAS associated genes. In other words, our study that analyzes expression data from MS patients focusing on changes not in the expression of single genes, but in gene connectivity, supports the evidence of association with MS resulting from SNP studies of genes that have not been highlighted by the previous differential expression analysis on the same data. Among the DC genes, the amiloride-sensitive cation channel 1 neuronal (*ACCN1*) presents significant changes in its mRNA expression in only one study, but exhibits a substantial reduction in the number of its interacting partners in the MS gene co-expression networks for all three independent studies. This gene has been associated with multiple sclerosis by using SNP studies [26,41] and encodes for a protein localized in the postsynaptic membrane that has a role in the neuronal transmission and the CNS development [93].

Finally, in our view, unveiling network properties that distinguish diseased from the healthy molecular states, as well as those that emerge in response to treatments allows one to gain systems-level insights into disease mechanisms and, ultimately, helps to identify novel molecular targets for specific treatments.

## 4. Materials and Methods

### 4.1. Data Collection

We searched the Gene Expression Omnibus (GEO) repository [16] for the most numerous datasets of the gene expression of MS patients and HCs. We selected the datasets in the GSE41850 SuperSeries ([17]) that includes 4 different datasets about two different comparisons MS *vs.* HC (GSE41848,GSE41849) and untreated *vs.* IFN-β treated MS patients (GSE41846, GSE41847). For each comparison, this series includes a discovery and a replication dataset (see Table 1). Moreover, we searched the GEO database for another independent dataset for each comparison with patients with similar characteristics to the patients enrolled in GSE41850 study. We selected the GSE17048 [18] dataset for comparing at the molecular level MS patients *vs.* HCs and the GSE16214 [19,20] dataset for studying changes in the gene expression profiles of MS patients treated with IFN-β. Table 6 shows that the different datasets are comparable in terms of the percentage of female, age, EDSS (Expanded Disability Status Scale) values and duration of disease. From these populations, we selected patients affected by RRMS, the most common clinical form of the disease. The GSE41850, the GSE17048 and the GSE16214 datasets were profiled by using the Affymetrix Human Exon 1.0 ST Arrays platform that measures 17,551 unique transcripts, by Illumina HumanHT-12 V3.0 expression BeadChip, that measures 13,162 unique transcripts, and by the Affymetrix Human Genome U133 Plus 2.0 Arrays, containing 54,675 probe sets, mapped on 19,520 unique genes, respectively. As all data files are available from the GEO repository, our study does not require an ethics statement. The data were preprocessed using the Affymetrix Expression Console: probe-level data in each tissue were background-adjusted, base 2 log transformed and normalized with the Robust Multi-array Average (RMA) procedure.

### 4.2. Data Analysis

#### 4.2.1. Gene Differential Expression

The gene differential expression (DE) *p*-values were evaluated by a two-tailed Student’s *t*-test, and the *p*-values were controlled for multiple testing using the Benjamini–Hochberg false discovery rate (FDR) [94].

#### 4.2.2. Network Inference

Given two microarray datasets from two different conditions, our approach of differential networking requires the inferring of the gene co-expression networks associated with the two different phenotypes. Each network is described by an undirected graph having a number of nodes equal to the number of assayed genes. Moreover, an edge linking a pair of nodes indicates a significant non-null co-expression between the two corresponding genes. As a co-expression measure between two genes, we adopted Spearman’s rank order correlation coefficient, because it takes into account some forms of non-linearity present in the data. The correlation *p*-values were computed using a normal distribution in the large sample approximation.

The assessment of the statistical significance *α* of the sample correlation coefficient is strongly influenced by the sample size. In a formula, in the large sample approximation, the significance of sample correlation *r* estimated from a sample of size *N* was assessed by considering the test statistic:

(1) $$t=\sqrt{\left(N-2\right)\cdot \frac{r^{2}}{1-r^{2}}}$$

which is *t*-distributed with (*N* − 2) degrees of freedom when the population correlation is null, *i.e.*,

(2) p-value=2T(-abs(t),N-2)

where *T* is the Student’s *t* cumulative distribution function. It follows that at a given level of statistical significance, the greater the sample size, the smaller is the minimum absolute value of the sample correlation coefficient that is significantly different from zero. As a consequence, at a fixed level of significance, in the case of samples from the same population, but of different sizes, it will result in a greater number of significant correlation coefficients in the more numerous sample. For this reason, in order to compare the gene degree distributions of the networks inferred in two different conditions, it is necessary to consider two samples of the same size. The GEO datasets under analysis include a different number of samples from healthy controls and multiple sclerosis patients. To address this issue, a strategy of random sampling was performed in order to make comparable the networks inferred in the two conditions. In detail, we performed 10 random sub-samplings of the more numerous sample in order to obtain 10 samples of the same size of the less numerous sample. For each GEO study, we repeated the entire analysis on the resulting 10 datasets that include an equal number of samples from both healthy controls and patients. This procedure has a two-fold advantage: it allows one to make comparable the disease and the healthy control networks and offers the possibility to increase the statistical power, giving more accurate results.

In order to infer the networks in the two conditions in each study and in each subsample, we set a link between two genes when the Spearman correlation coefficient between their expression profiles resulted in being greater than 0.7 [95]. The different GEO studies consider samples of different sizes. Due to the influence of sample size on the statistical significance of the sample correlation coefficient, a different level of *α* was adopted for each study. From Equation (2), it follows that, at a fixed threshold for the correlation coefficient, the greater is the sample size, the smaller is the level of significance *α*, *i.e.*, the *p*-value of the minimum sample correlation coefficient that can be considered significantly different from zero. In detail, it results in a value of *α* that varies from 1 × 10 ${}^{-3}$ to 2 × 10 ${}^{-6}$ in the case of the sample sizes of 15 and 36, respectively. This procedure allows also to check if the number of samples is large enough to ensure an acceptable level of significance.

#### 4.2.3. Gene Differential Connectivity

For each gene, we calculated its degree or connectivity, which is defined as the number of vertices connected to it in the network. From a biological point of view, in a co-expression network, the degree of a given gene quantifies the number of genes “co-expressed” with it. Then, we calculated the absolute value of the difference between the degrees in the two different phenotypes [12]. To assess the statistical significance of this value, we used a non-parametric permutation test. We randomly assigned the gene expression profiles of the samples to two subsets independently of their phenotype and repeated the analysis. This was done 1000 times to evaluate the null distribution of the degree difference under the hypothesis of any association between expression profile and phenotype. Real degree differences were compared to the null distribution to evaluate *p*-values. We addressed the multiple hypothesis correction by controlling the FDR. We called differentially connected (DC) those genes having *p*-values below 0.05.

#### 4.2.4. Meta-Analysis of DC *p*-Values

The random sampling procedure described in the Network Inference section generates for each gene and for each study a list of ten DC *p*-values, which measure the association level of that gene with the phenotype. In order to estimate unique values for the DC statistics and *p*-values, we adopted a meta-analysis procedure based on the assumption that a gene is considered DC only if this property is confirmed in more experiments. At a given level of significance, the higher the number of times *K* a gene results in being statistically significant, the more such an association is not due to chance. However, what is the minimum number of times *K* we have to find a gene DC in order to conclude that it is a DC gene? The $K_{min}$ can be chosen as the *K* that corresponds to a significant consistency among the findings of the different experiments. To this aim, it is necessary to estimate a false discovery rate associated with the number *K* in order to control the expected proportion of incorrectly-rejected null hypotheses. It is important to underline that the significance of *K* depends on the number of genes resulting in DC in each experiment, because the greater the number of DC genes in each list, the greater is the number of times that a gene results in DC also in the case of independent lists, *i.e.*, in the case of lists of inconsistent findings. We designed a procedure to address this issue regarding it as an extension of Fisher’s exact test when the number of conditions to be compared is greater than two. In particular, we counted the number of times *K* each gene results in being statistically significant and evaluated the probability to obtain a number equal to or greater than *K* in the case that the lists are independent of each other. In other words, a *p*-value was associated with each *K* in {0, ..., 10} by considering the null distribution of *K* as the binomial B(K,10,p) where *p* is the probability that a gene results in DC at the given level of significance in a single experiment. We adopted a conservative choice and estimated *p* as the maximum of the fractions of the resulting DC genes in each experiment. In a formula, given $n_{DC}^{i}$ the number of DC genes in *i*-th experiment and *n* the number of assayed genes, one defines:

(3) $$P-value\left(K\right)=\sum_{x=K}^{10}B\left(x,10,p\right)$$

where $p=\max_{i=1,...,10}\frac{n_{DC}^{i}}{n}$. To each gene, we associated the value of *K* and the corresponding *p*-value, and the multiple testing was controlled by applying the Benjamini–Hochberg false discovery rate (FDR) algorithm [94]. Fixing a threshold for FDR of 0.05, we chose for each dataset the minimum number of times $K_{min}$ we have to find a gene DC. To each gene, we associated a DC *p*-value, which is the maximum *p*-value among the first $K_{min}$ smallest *p*-values and the differential connectivity statistics as the median on the corresponding degree differences.

#### 4.2.5. Gene-Set Enrichment of SNPs by a Genome-Wide Association Study

We retrieved from the NCBI dbGaPdatabase the GWAS results of the phs000171.v1.p1 record. This entry collects the results of the study published in the paper [41]. Starting from the *p*-values for association with MS of each SNP, we computed a *p*-value of association for each gene. By MATLAB codes included in the MAGENTA software package, we mapped SNPs onto genes. Gene boundaries used for mapping SNPs are: 110 kb upstream to the most extreme gene transcript start position and 40 kb downstream to the most extreme gene transcript end position, taking gene orientation into account. Note that the adopted gene boundaries are the default parameters in the MAGENTA software [96]. Then, we searched for the SNP with the minimum *p*-value for each gene and associated this *p*-value with the gene. By a Fisher’s exact test, we tested the lists of DE and DC genes that are significantly enriched with genes with GWAS *p*-values ≤ 0.006.

#### 4.2.6. Differential Expression and Connectivity Gene Set Enrichment Analysis

An enrichment pathway analysis was performed in order to reveal among a set of predefined gene sets those sets that are significantly enriched with DE and DC genes, respectively. The *p*-values for the enrichment of these gene sets were evaluated by using the Compute Overlaps MsigDB tool based on Fisher’s exact test [21]. We analyzed gene sets corresponding to the biological processes, the molecular functions and the cellular components in the Gene Ontology collection and the gene sets of the canonical pathway collection (C2CP), including categories from the Reactome, KEGG and Biocarta databases retrieved from MsigDB [21]. The Compute Overlaps MsigDB tool was used also to identify the overrepresented transcription factor binding sites in the promoter regions and microRNA binding motifs in the 3’-UTR of the DE and DC genes. The lists of genes sharing a specific transcription factor binding site in their promoter regions and those of genes sharing a microRNA binding motif in their 3’-UTR were retrieved from MsigDB. The algorithms here described were implemented in the MATLAB programming language.

## Figures and Tables

**Table 1:** Main features of the gene expression arrays.

Reference	[17]						[18]		[19,20]
GEO datasets - SuperSeries	GSE41850						GSE17048		GSE16214
**Platform**	Affymetrix Human Exon 1.0 ST Array						Illumina HumanHT-12 V3.0 expression beadchip		Affymetrix Human Genome U133 Plus 2.0 Array
**Tissue**	Whole blood						Whole blood		Peripheral blood mononuclear cells
**Total genes studied**	17,551						13,162		19,520
**GEO datasets - SubSeries**	**Discovery sets**			**Replications sets**					
	**GSE41846**	**GSE41848**		**GSE41847**	**GSE41849**				
**Comparisons**	**Untreated *vs.* IFN β-treated**	**MS *vs.* HC**		**Untreated vs IFN β-treated**	**MS *vs.* HC**		**MS *vs.* HC**		**Untreated *vs.* IFN β-treated**
**Condition of the analyzed arrays**	**MS**	**MS**	**HC**	**MS**	**MS**	**HC**	**MS**	**HC**	**MS**
**Number of the analyzed arrays**	98	37	79	63	15	46	36	45	176
**Untreated/IFN β-treated**	37/61	37/0	-	15/48	15/0	-	36/0	-	82/94

**Legend**: MS = multiple sclerosis; HC = healthy controls.

**Table 2:** Number of differentially-expressed (DE) and differentially-connected (DC) genes for each analyzed dataset and confirmed in 2 or 3 experiments. Note that in the column entitled “Genes”, for the genes confirmed in more experiments, we reported the number of common genes among the different platforms.

Study	Genes	DE	DC	Reference
GSE41848	17,551	1520	741	[17]
GSE41849	17,551	2164	449	[17]
GSE17048	13,162	272	1223	[18]
GSE41846	17,551	1358	759	[17]
GSE41847	17,551	631	207	[17]
GSE16214	19,520	1522	790	[19,20]
MS *vs.* HC (in 2 of 3 datasets)	7941	227	29	[17,18]
MS *vs.* HC (in 3 datasets)	7941	12	4	[17,18]
IFN treated *vs*. untreated (in 2 of 3 datasets)	14,794	563	60	[17,19,20]
IFN treated *vs*. untreated (in 3 datasets)	14,794	188	15	[17,19,20]

**Table 3:** Genes DC at the level of 0.05 in the MS *vs.* HC comparison in at least 2 studies.

Gene Symbol	Chr	Gene Name	Function	Reports in MS	Other Associations	References
					with CNS Diseases	
*ACCN1*	17	acid sensing (proton gated)	ion transport, neuronal transmission,	yes		[26,27]
		ion channel 2 (*ASICS2*)	CNS development			
*OR7A17*	19	olfactory receptor, family 7,	signal transducer activity			
		subfamily A, member 17				
*FH*	1	fumarate hydratase	protein binding, lyase activity		Parkinson’s disease	[28]
*GJA5*	1	gap junction protein, alpha 5, 40 kDa	cell communication, component		angiogenesis	
			of plasma membrane			
*PAIP1*	5	poly(A) binding protein interacting protein 1	RNA and protein binding		FALS	[29]
*MLL4*	12	lysine (K)-specific methyltransferase 2D (KMT2D)	transcription regulatory DNA		developmental brain	[30]
*DNAJC2*	7	DnaJ (Hsp40) homolog, subfamily C, member 2	ubiquitin and histone binding			
*KIF18A*	11	kinesin family member 18A	microtubule binding			
*UBE2H*	7	ubiquitin-conjugating enzyme E2H	ubiquitin-dependent protein catabolic processes		autism	[31]
*FLRT3*	20	fibronectin leucine-rich transmembrane protein 3	cell adhesion, axonal guidance,		*ADHD*	[32]
			neuron projectiondevelopment			
*GLS*	2	glutaminase	neurotransmitter secretion, synaptic transmission	yes		[33]
*LMBR1L*	12	limb development membrane protein 1-like	endocytosis processes			
*KRTAP5-4*	11	keratin-associated protein 5-4	component of keratin filament			
*PRKD2*	19	protein kinase D2	adhesion, cell death, immunity		glioma	[34]
*GABRR2*	6	gamma-aminobutyric acid (GABA) A receptor, rho 2	GABA-receptor activity, synapses component		epilepsy	[35]
*HIST2H2AB*	1	histone cluster 2, H2ab	chromatin organization and silencing			
*PDE6D*	2	phosphodiesterase 6D, cGMP-specific, rod, delta	visual perception, cell projection			

**Table 4:** Genes DC at the level of 0.05 in the MS *vs.* HC comparison in at least 2 studies.

Gene Symbol	Chr	Gene Name	Function	Reports in MS	Other Associations	Reference
					with CNS Diseases	
*DLG1*	3	discs, large homolog 1	axon guidance, regulation of		Parkinson’s disease	[36]
			myelination, immunological synapses			
*EYA3*	1	EYA transcriptional co-activator	histone dephosphorylation, DNA repair			
		and phosphatase 3				
*PPA2*	4	pyrophosphatase (inorganic) 2	mitochondrial matrix component			
*SPTA1*	1	spectrin, alpha, erythrocytic 1	axon guidance, innate immune response			
*ELSPBP1*	19	epididymal sperm binding protein 1	component of extracellular regions			
*PRKAR2A*	3	protein kinase, cAMP-dependent,	innate immune response		Lewis body diseases	[37]
		regulatory, type II, alpha				
*TCP1*	6	t-complex 1	myelin sheath component,		epilepsy	[38]
			DNA and protein binding			
*KHDRBS2*	6	KH domain containing, RNA binding,	protein heterodimerization activity		Alzheimer Disease	[39]
		signal transduction associated 2				
*TRIM35*	8	tripartite motif containing 35	apoptosis, immunity			
*SIKE1*	1	suppressor of IKBKE 1	immunity, cell proliferation			
*HDAC2*	6	histone deacetylase 2	transcriptional regulation, cell cycle	yes		[40]
			progression and developmental events			
*RNASE11*	14	ribonuclease, RNase A family, 11	hydrolase activity			

**Table 5:** Genes DC at the level of 0.05 in the untreated *vs.* IFN-β treated comparison in all 3 studies.

Gene Symbol	Chr	Gene Name	Function	Reports in MS	Other Associations	Reference
					with CNS Diseases	
*SPATS2L*	2	spermatogenesis associated, serine-rich 2-like	protein binding, poly(A) RNA binding			[47,48]
*LAMP3*	3	lysosomal-associated membrane protein 3	dendritic cell function, in adaptive immunity	yes		[49,50]
*ETV7*	6	Etsvariant 7	transcriptional repressor activity, sequence-specific	yes		[51]
			DNA binding, protein binding			
*TRIM14*	14	tripartite motif containing 14	protein binding, zinc ion binding			
*USP18*	22	ubiquitin-specific peptidase 18	cysteine-type endopeptidase activity,	yes		[52]
			ubiquitin-specific protease activity, protein binding			
*OASL*	12	2’-5’-oligoadenylate synthetase-like	DNA, RNA and protein binding,	yes		[19]
			thyroid hormone receptor binding			
*MOV10*	1	Mov10 RISC complex RNA helicase	helicase activity, protein and ATP binding		synaptic plasticity	[53,54]
*OTOF*	2	otoferlin	calcium ion binding, protein binding,		autism depressive disorder	[55,56]
			AP-2 adaptor complex binding			
*GBP1*	1	guanylate binding protein 1,	GTPase activity, protein binding	yes		[57]
		Interferon-Inducible				
*XAF1*	17	XIAP-associated factor 1	protein binding, zinc ion binding	yes		[58]
*LY6E*	8	lymphocyte antigen 6 complex, locus E	signal transduction	yes		[59]
*RSAD2*	2	radical S-adenosyl	catalytic activity, protein binding,	yes		[17]
		methionine domain containing 2	metal ion binding			
*ISG15*	1	ISG15 ubiquitin-like modifier	protein binding, protein tag	yes		[60]
*OAS3*	12	2’-5’-oligoadenylate synthetase 3	RNA binding, protein binding,	yes		[61]
			ATP binding, synthetase activity			
*DHX58*	17	DEXH (Asp-Glu-X-His) box polypeptide 58	DNA, RNA and protein binding, helicase activity	yes		[62]

**Table 6:** Main features of the clinical/demographic characteristics of the related populations.

Reference	[17]		[17]		[18]		[19,20]
GEO datasets	GSE41846-GSE41848		GSE41847-GSE41849		GSE17048		GSE16214
	Discovery sets		Replications sets				
	MS	HC	MS	HC	MS	HC	MS
**Number of the recruited subjects**	98	41	63	25	36	45	176
**Untreated/IFN β-treated**	37/61	-	15/48	-	36/0	-	82/94
**% Female**	70	75	65	55	81	64	82
**Clinical MS forms (CIS/RR/SP/PP in %)**	13/80/7/0	-	11/87/2/0	-	0/100/0/0	-	5/95/0/0
**Age at MS onset (mean; range)**	-	-	-	-	33 (19-56)	-	36 (17-63)
**Disease duration (mean/median; range/SD)**	8 (2-47)	-	6 (2-27)	-	15 (1-36)	-	8 (0-43)
**EDSS (mean/median; range/SD)**	2 (0-7)	-	2 (0-6)	-	2.7 (0-6.5)	-	-
**Age (median/mean + range; mean + SD)**	45 (24-66)	46 (26-66)	46 (25-61)	42 (27-61)	48.5 (29-56)	48.5 (23-77)	-

**Legend**: MS = multiple sclerosis; HC = healthy controls; CIS = clinically isolated syndrome; RR = relapsing remitting; SP = secondary progressive; PP = primary progressive.

## Supplementary Materials

Supplementary materials can be found at http://www.mdpi.com/1422-0067/17/ 6/936/s1: Table S1: DE genes in MS. List of DE genes with *p*-value ≤0.01 in at least two of the datasets relative to the comparison of MS patients *vs.* HCs. Table S2: DC genes in MS. List of DC genes with *p*-value ≤0.05 in at least two of the datasets relative to the comparison of MS patients *vs.* HCs. Table S3: DE genes in IFN treated patients. List of DE genes with *p*-value ≤0.01 in at least two of the datasets relative to the comparison of IFN treated *vs.* untreated MS patients. Table S4: DC genes in IFN treated patients. List of DC genes with a *p*-value ≤0.05 in at least two of the datasets relative to the comparison of IFN treated *vs.* untreated MS patients. Table S5: DE pathways in MS patients. List of canonical pathways, GO terms, transcription factor and microRNA binding motifs over-represented in the list of the DE genes in MS patients in at least one study. Table S6: DC pathways in MS patients. List of canonical pathways, GO terms, transcription factor and microRNA binding motifs over-represented in the list of the DC genes in MS patients in at least one study. Table S7: DE pathways in IFN-treated patients. List of canonical pathways, GO terms, transcription factor and microRNA binding motifs over-represented in the list of the DE genes in IFN-treated patients in at least one study. Table S8: DC pathways in IFN-treated patients. Supplementary Table 8: List of canonical pathways, GO terms, transcription factor and microRNA binding motifs over-represented in the list of the DC genes in IFN-treated patients in at least one study. MATLAB Code: MATLAB codes used for the analysis of differential connectivity.

## Abbreviations

MS: Multiple Sclerosis
IFN: Interferon
HC: Healthy condition
DE: Differential expression
DC: Differentially connected
FDR: False discovery rate
DCA: Differential co-expression analysis
EAE: Transcription factor binding site
TFBS: Differential expression
CNS: Central nervous system
SNP: Single-nucleotide polymorphisms
GWAS: Genome-wide association study
HLA: Human leukocyte antigen
